# A Spiking Neurocomputational Model of High-Frequency Oscillatory Brain Responses to Words and Pseudowords

**DOI:** 10.3389/fncom.2016.00145

**Published:** 2017-01-18

**Authors:** Max Garagnani, Guglielmo Lucchese, Rosario Tomasello, Thomas Wennekers, Friedemann Pulvermüller

**Affiliations:** ^1^Department of Computing, Goldsmiths, University of LondonLondon, UK; ^2^Brain Language Laboratory, Department of Philosophy and Humanities, Freie Universität BerlinBerlin, Germany; ^3^Berlin School of Mind and Brain, Humboldt Universität zu BerlinBerlin, Germany; ^4^Centre for Robotics and Neural Systems, University of PlymouthPlymouth, UK

**Keywords:** neural network, cell assembly, gamma band, language, synchrony, simulation, Hebbian learning

## Abstract

Experimental evidence indicates that neurophysiological responses to well-known meaningful sensory items and symbols (such as familiar objects, faces, or words) differ from those to matched but novel and senseless materials (unknown objects, scrambled faces, and pseudowords). Spectral responses in the high beta- and gamma-band have been observed to be generally stronger to familiar stimuli than to unfamiliar ones. These differences have been hypothesized to be caused by the activation of distributed neuronal circuits or cell assemblies, which act as long-term memory traces for learned familiar items only. Here, we simulated word learning using a biologically constrained neurocomputational model of the left-hemispheric cortical areas known to be relevant for language and conceptual processing. The 12-area spiking neural-network architecture implemented replicates physiological and connectivity features of primary, secondary, and higher-association cortices in the frontal, temporal, and occipital lobes of the human brain. We simulated elementary aspects of word learning in it, focussing specifically on semantic grounding in action and perception. As a result of spike-driven Hebbian synaptic plasticity mechanisms, distributed, stimulus-specific cell-assembly (CA) circuits spontaneously emerged in the network. After training, presentation of one of the learned “word” forms to the model correlate of primary auditory cortex induced periodic bursts of activity within the corresponding CA, leading to oscillatory phenomena in the entire network and spontaneous across-area neural synchronization. Crucially, Morlet wavelet analysis of the network's responses recorded during presentation of learned meaningful “word” and novel, senseless “pseudoword” patterns revealed stronger induced spectral power in the gamma-band for the former than the latter, closely mirroring differences found in neurophysiological data. Furthermore, coherence analysis of the simulated responses uncovered dissociated category specific patterns of synchronous oscillations in distant cortical areas, including indirectly connected primary sensorimotor areas. Bridging the gap between cellular-level mechanisms, neuronal-population behavior, and cognitive function, the present model constitutes the first spiking, neurobiologically, and anatomically realistic model able to explain high-frequency oscillatory phenomena indexing language processing on the basis of dynamics and competitive interactions of distributed cell-assembly circuits which emerge in the brain as a result of Hebbian learning and sensorimotor experience.

## Introduction

Experimental evidence suggests that the cortex stores knowledge about meaningful, well-known familiar items (such as objects, faces, and words) as distributed memory circuits, that is, strongly interlinked neuronal ensembles of hundreds or thousands of neurons whose members may be spread across distant areas of cortex. The reactivation of such a cell assembly (CA) circuit sparked by the perception of the corresponding sensory item is hypothesized to induce waves of reverberant activity within the corresponding circuit (Hebb, 1949), measurable as correlated firing activity. Intracortical recordings of stronger high-frequency synchronous oscillations during perception of coherent vs. incoherent visual stimuli were thus taken as crucial support for the existence of such mutually supporting neuronal ensembles in the brain (Singer, 1993; Singer and Gray, 1995; Engel and Singer, 2001; Varela et al., 2001; Buzsáki and Draguhn, 2004). In the cognitive domain, observed increases in the oscillatory cortical responses to meaningful, well-known stimuli compared to senseless, unknown sensory material also provide evidence for the existence of stimulus-specific memory traces for frequently occurring percepts (and lack thereof for novel, unfamiliar ones) (Pulvermüller et al., 1994; Krause et al., 1998; Henson et al., 2009; Tallon-Baudry, 2009; Hassler et al., 2011; Bertrand et al., 2013; Craddock et al., 2015). The majority of experiments testing this hypothesis focus on fast oscillatory activity, even though other types of correlation can also exist (Abeles, 1991). In particular, differences in spectral responses have typically been found in the so-called gamma band (around 40 Hz), but also in the low-gamma and high-beta (20–30 Hz) and very high gamma (above 100 Hz) bands, across different modalities and using different recording methods. In the visual domain, the role of gamma-band activity has been intensively researched: a number of studies have reported differences in oscillatory responses to recognizable, coherent, complete, meaningful stimuli vs. unrecognizable, scrambled, incoherent or incomplete visual ones, including, e.g., real or illusory (Kanizsa) triangle and no-triangle (Tallon-Baudry et al., 1996), pictures and fragmented images (Gruber et al., 2002; Bertrand et al., 2013), objects and non-objects (Craddock et al., 2015), and faces vs. scrambled faces (Henson et al., 2009; Gao et al., 2013). Notably, only responses to the coherent stimuli have been found to induce synchronous oscillations across neurons located in different cortical hemispheres (Supp et al., 2005, 2007).

High-frequency dynamics like gamma oscillations have been implied in the recognition of familiar sensory items also in the language domain, with meaningful words consistently inducing stronger spectral responses than senseless, unknown pseudoword items for frequencies between 20 and 40 Hz (Lutzenberger et al., 1994a; Eulitz et al., 1996; Pulvermüller et al., 1996a; Krause et al., 1998), and, occasionally, even in higher frequency ranges (up to 200 Hz: Canolty et al., 2007; Mainy et al., 2008). Some studies suggested that aspect of the meaning of words might be reflected in different high-frequency response topographies and long-range gamma synchrony across the cortex (Pulvermüller et al., 1996b; Weiss and Müller, 2013); the suggestion here was that the underlying neuronal circuits carrying words and their meaning might be differentially distributed across cortical areas depending on the semantic category of the stimulus.

We focus here on the manifestation of the above-mentioned differences in oscillatory behavior as observed in the linguistic domain. In particular, the main goal of the present study is to reproduce the neurophysiological findings of larger spectral power for words than pseudowords observed in the 20–40 Hz range using a neuroanatomically realistic computational model of the cortex, and examine the model's behavior at the cortical-circuit level to shed some light on the underlying neural mechanisms. Recent simulation results obtained using biologically realistic models of the left-perisylvian (“language”) cortex similar to the one used here have mechanistically demonstrated the spontaneous formation and activation dynamics of distributed memory circuits for words, which emerged in the network as a result of Hebbian learning (Hebb, 1949) and simulated “sensorimotor” experience (Garagnani et al., 2007, 2008; Garagnani and Pulvermüller, 2011, 2016; Tomasello et al., 2016). Our hypothesis was that, if the difference in high-frequency responses induced by familiar vs. unfamiliar items can be related to the presence of memory traces for the former and absence thereof for the latter, the same computational model should be able to reproduce the above-mentioned experimental findings, potentially providing an explanatory account for the enhanced high-frequency brain responses to lexical items on the basis of the activation of such stimulus-specific cell-assembly (CA) circuits.

Gamma oscillations and their synchronization have been investigated computationally and theoretically in numerous studies (see Wang, 2010; Buzsáki and Wang, 2012 for reviews). Oscillations easily occur in simulations of networks of spiking neurons, regardless of whether these are made up of simple leaky integrate-and-fire (LIF) cells or more complex neuron types (e.g., Traub et al., 2000; Sommer and Wennekers, 2001; Izhikevich and Edelman, 2008; Herman et al., 2013). Various mechanisms for the origin of oscillations in the gamma range are known: Brunel (2000), for example, has mathematically analyzed the quite generic case of two pools of excitatory and inhibitory LIF neurons. While the use of excitatory and inhibitory populations is very common in computational studies (including the present one) further mechanisms have been also proposed as potential sources of cortical gamma oscillations, such as synaptic inhibition and correlation-induced stochastic synchrony (Wang, 2010; Whittington et al., 2011).

A variety of localist and distributed connectionist models have been proposed in the past to explain the putative mechanisms underlying speech processes (e.g., McClelland and Elman, 1986; Seidenberg and McClelland, 1989; Norris, 1994; Seidenberg et al., 1994; Gaskell et al., 1995; Plaut et al., 1996; Dell et al., 1997; Page, 2000; Rogers et al., 2004; see Woollams, 2015 for a recent review). One of the earliest, most influential connectionist models of memory (McClelland and Rumelhart, 1985), for example, was able to account for basic differences in repetition priming of spoken words and pseudowords (a word being represented as a distributed pattern of activity across a layer of units). Nowadays, a new generation of large-scale neural-network models are being increasingly used in the study of memory and language processes (e.g., Wennekers et al., 2006; Herman et al., 2013; Pulvermüller and Garagnani, 2014; Hinaut et al., 2015; Rolls and Deco, 2015), which are able to elucidate the underlying brain mechanisms on the basis of neurobiologically realistic learning and anatomical connectivity, and explain neuroimaging data (Husain et al., 2004; Pulvermüller et al., 2014; Garagnani and Pulvermüller, 2016). However, to date, a neuromechanistic account directly linking the different high-frequency neurophysiological responses induced by familiar word and unknown pseudoword stimuli to corresponding differential oscillatory behavior of underlying large-scale neuronal populations is still missing.

## Materials and methods

### General structure and features of the model

**Figure 1:**
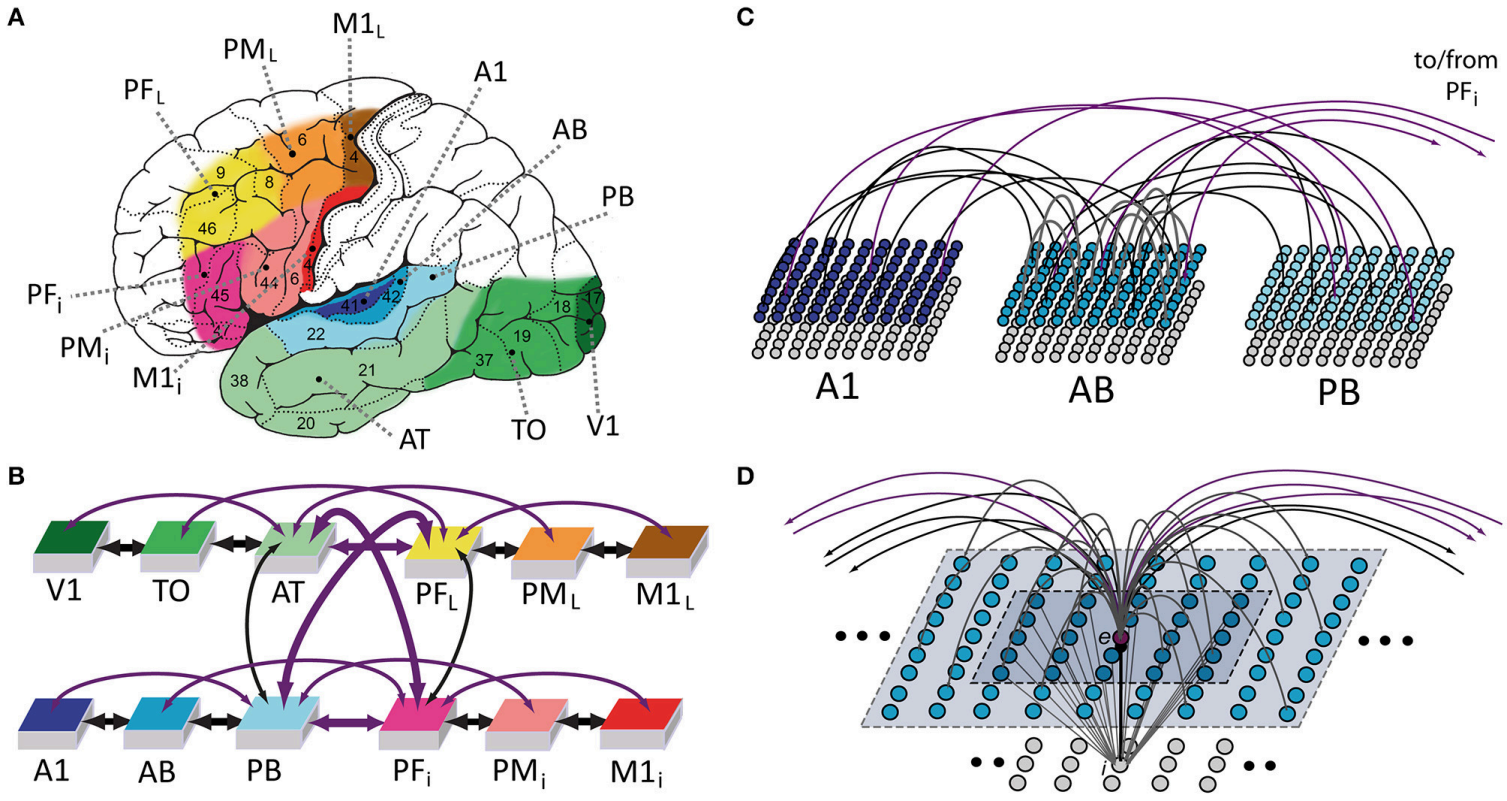
**Model of lexical and semantic mechanisms: the 12 cortical areas simulated (A)**, their global connectivity architecture **(B)**, aspects of between- **(C)** and within-area **(D)** connectivity of model neurons are illustrated. **(A)** Six perisylvian and six extrasylvian areas are shown, each including an anterior (frontal) and a posterior (temporal) part. Perisylvian areas include three areas in inferior frontal gyrus (colored in different shades of red), including inferior-prefrontal (PF_i_), premotor (PM_i_), and primary motor cortex (M1i) and three areas in the superior temporal lobe (shades of blue), including auditory parabelt (PB), auditory belt (AB), and primary auditory cortex (A1). These areas are relevant for storing and linking up articulatory-phonological and corresponding acoustic-phonological patterns of neuronal activations, which co-occur, for example, when spoken word forms are being articulated (activity in M1i) and corresponding speech sounds are simultaneously perceived (stimulation of primary auditory cortex, A1). Extrasylvian areas include three areas in lateral/superior frontal cortex (yellow to brown), including dorsolateral prefrontal (PF_L_), premotor (PM_L_), and primary motor cortex (M1_L_) and three areas forming the occipito-temporal (“what”) visual stream of object processing (different shades of green), including anterior-temporal (AT), temporo-occipital (TO), and early visual areas (V1). These areas contain neural patterns carrying semantic information (word meaning), for example when words are used (activity in all perisylvian areas) to talk about objects present in the environment (activity in V1, TO, AT) or about actions currently being performed (activity in M1_L_, PM_L_, PF_L_). Numbers indicate Brodmann Areas (BAs). **(B)** Schematic illustration of the 12 modeled areas and between-area connections implemented (shown as bidirectional arrows). The colors indicate the correspondence between cortical and model areas. Thick and thin arrows indicate links already implemented in previous mean-field versions of the architecture and newly added ones, respectively. Arrow color discriminates “next-neighbor” connections (in black), linking cortically adjacent areas, from “jumping” ones (in purple), between non-adjacent cortical areas. See main text for the neuroanatomical evidence used to determine the model's connectivity structure. **(C)** Schematic illustration of connectivity between three areas of the model. Each area consists of two layers (or banks) of 25 × 25 excitatory (upper) and inhibitory (lower) integrate-and-fire cells exhibiting neuronal fatigue. Between-area connections (black and purple) are sparse, random and topographic. **(D)** Neuron-level connectivity of one of the 7500 single excitatory neural elements modeled (labeled “*e*”). Within-area excitatory links (in gray) to and from “cell” *e* are random and sparse, and limited to a local (19 × 19) neighborhood of neural elements (area shaded in light-blue). Lateral inhibition between *e* and neighboring excitatory elements is realized as follows: the underlying cell “*i*” inhibits e in proportion to the total excitatory input it receives from the 5 × 5 neighborhood (darker-blue shaded area); by means of analogous connections (not depicted), *e* inhibits all of its neighbors.

We used a neural-network architecture to simulate cortical mechanisms underlying language function in the left hemisphere of the human brain (Figure 1A). The network is divided into twelve identical “areas” of spiking artificial neurons with reciprocal connections between and within them (see Figures 1B,C). Each area consists of two “banks” or layers of excitatory and inhibitory cells. The model was constructed so as to reflect a range of properties of the human cortex; the main features included: (1) local (see Figure 1D) and area-specific global inhibitory mechanisms (Braitenberg, 1978b; Yuille and Geiger, 2003); (2) patchy, random and topographic connections, with probability of a synaptic link being established between two cells decreasing with their distance (Kaas, 1997; Braitenberg and Schüz, 1998); (3) presence of uniform noise (simulating spontaneous, baseline neuronal firing) in all network areas at all times (Rolls and Deco, 2010); and (4) Hebbian synaptic plasticity, simulating well-known phenomena of long-term potentiation (LTP) and depression (LTD) (Artola and Singer, 1993). These features are identical to those used in our previous versions of the architecture (Garagnani et al., 2008; Garagnani and Pulvermüller, 2011, 2013, 2016). Excitatory neurons are now modeled as leaky integrate-and-fire cells with adaptation, whereas our previous simulations used a “lumped” or mean-field approach, with each cell representing the average activity of a local pool or cluster of neurons (Wilson and Cowan, 1973; Eggert and van Hemmen, 2000). In line with the introduction of spiking cells, the present model also implements a revised version of Hebbian learning, in which the presence of a pre- or post-synaptic spike is a necessary (but not sufficient) pre-requisite for any synaptic changes to take place. The full formal specification of the model is provided in Section Model Specification below.

During speech production, patterns of neural activity co-occur in primary motor and auditory cortices as a consequence of the articulatory movements and simultaneous perception of the corresponding uttered sounds; hence, both of these primary perisylvian areas (labeled M1i and A1, respectively) were modeled. Furthermore, as the processing of information about the referential meaning of object-related words (such as “flower”) involves primary visual cortex, and because execution of the action corresponding to the meaning of words such as “run” or “grasp” is controlled by the more lateral and superior aspects of the motor cortex, the model also included primary visual (V1) and dorsolateral motor (M1_L_) cortices (see Figure 1A). In addition to these four primary areas, “higher” secondary and multimodal cortices which are known to have direct anatomical links with the above four primary sensorimotor cortices were also included (see below for details and supporting neuroanatomical evidence). These were secondary inferotemporo-occipital visual, auditory belt, and inferior and lateral premotor cortex (TO, AB, PM_i_, PM_L_) and, respectively, adjacent multimodal anterior-temporal, superior-temporal (auditory parabelt) and inferior and dorsolateral prefrontal cortices (AT, PB, PF_i_, PF_L_).

The present model builds upon and extends an existing architecture recently used to simulate neural mechanisms underlying acquisition of action- and visually-related words (Garagnani and Pulvermüller, 2016; Tomasello et al., 2016). The present study further augments the architecture by introducing (i) additional between-area connections and (ii) spiking artificial neurons. This level of granularity was deemed appropriate to simulate the phenomena of interest here, namely, the spontaneous emergence of synchronous oscillations in cortically distributed neuronal populations.

As in all previous versions, we strived to implement only mechanisms reflecting well-documented neurophysiological phenomena. Crucially, the network's connectivity structure (depicted by black and purple bidirectional “arrows” in Figure 1B) closely reflects existing anatomical pathways between corresponding areas of the cortex, with between- and within-area synaptic projections mimicking known properties of the mammalian brain. The previous mean-field versions of the architecture (Garagnani and Pulvermüller, 2016) only realized a subset (thick arrows in Figure 1B) of the connections implemented here, that is, reciprocal links between next-neighbor areas within each triplet of the four “modality-specific” sub-systems modeled (thick black arrows in Figure 1B), and reciprocal links between anterior temporal (AT), superior parabelt (PB) and inferior (PF_i_), and superior-lateral (PF_L_) prefrontal areas (thick purple arrows in Figure 1B). The neuroanatomical evidence documenting presence of such links is reported in **Appendix A**. In addition to these, the following between-area anatomical connections are also modeled in the present version (thin arrows in Figure 1B):

- links between non-adjacent areas within the superior- or inferior temporal, superior or inferior frontal, cortices (i.e., within-modality “jumping” links), connecting primary auditory (A1) with parabelt (PB) areas (Pandya and Yeterian, 1985; Young et al., 1994), lateral/inferior prefrontal (PF_L_/i) with corresponding primary motor areas (M1_L_/i) (Deacon, 1992; Young et al., 1995; Guye et al., 2003), and primary visual (V1) with anterior temporal (AT) cortices (Catani et al., 2003; Wakana et al., 2004);
- long-distance connections between “auditory” (superior temporal gyrus) and “articulatory” (inferior frontal gyrus) perisylvian regions—specifically, linking parabelt (PB) with inferior premotor (PM_i_) areas (Glasser and Rilling, 2008; Saur et al., 2008, 2010; Petrides and Pandya, 2009) and belt (AB) with inferior prefrontal (PF_i_) areas (Romanski et al., 1999a; Kaas and Hackett, 2000; Rauschecker and Scott, 2009);
- long-distance links between extrasylvian “visual” (inferior-temporal, TO, AT) and “motor” (dorsolateral prefrontal and premotor, PF_L_/PM_L_) cortices, analogous to those listed above for the perisylvian (“auditory” and “articulatory”) systems, documented by both neuroanatomical (Pandya and Barnes, 1987; Seltzer and Pandya, 1989; Makris and Pandya, 2009) and inactivation studies in the macaque monkey (Bauer and Fuster, 1976, 1978; Fuster and Jervey, 1981; Fuster et al., 1985; Chafee and Goldman-Rakic, 2000).

Results from previous simulation studies have shown that, when repeatedly confronted with activity patterns to their “primary” (input) areas, networks including the above range of neurobiologically realistic features exhibit spontaneous formation of distributed associative circuits (Garagnani et al., 2007, 2008, 2009; Garagnani and Pulvermüller, 2016; Tomasello et al., 2016), or “cell assemblies” (CAs) (Hebb, 1949), networks of cells binding together patterns of frequently co-active neurons (Hebb, 1949; Braitenberg, 1978a; Palm, 1982). These circuits, which emerge as a result of correlational learning mechanisms, exhibit non-linear functional behavior, with two quasi-stable states (“on” and “off”) (Garagnani et al., 2007, 2008, 2009; Pulvermüller and Garagnani, 2014).

The previous, mean-field versions of the architecture exhibited the spontaneous formation of such lexico-semantic circuits in the context of simulated acquisition of object- and action-related words (Garagnani and Pulvermüller, 2016; Tomasello et al., 2016). In particular, the resulting CAs showed category-specific distributions, linking up “auditory-articulatory” patterns (simulating neural activity induced in M1i by word production and correlated activity in A1 due to perception of the corresponding sound) with semantic information present either in the model's perceptual (V1, object words) or motor (M1_L_, action words) areas. During simulated word-comprehension processes, reactivation of these circuits sparked the model's primary sensorimotor areas in a category-specific fashion, reflecting the patterns of activity that occurred in the network at word-learning stages (Garagnani and Pulvermüller, 2016; Tomasello et al., 2016).

Here, we trained the network following the same procedure used in the previous studies; as a result of the learning mechanisms, similarly distributed CAs emerged in this extended spiking architecture. After training, we recorded and analyzed the network's oscillatory responses to learned, meaningful, “word” patterns and novel, meaningless “pseudoword” stimuli (see Section Simulating Learning of Meaningful Words below), with a view to shed some light on the neuromechanistic causes underlying experimentally observed differences.

### Model specification

Each of the 12 simulated areas is implemented as two layers of artificial neuron-like elements (“cells”), 625 excitatory and 625 inhibitory, thus resulting in 15,000 cells in total (see Figures 1B,C). Each excitatory cell “*e*” consists of an integrate-and-fire neuron with adaptation and simulates a single pyramidal cell, while its twin inhibitory cell “*i*” (see Figure 1D) is a graded-response cell simulating the inhibitory response of the cluster of interneurons situated within the same cortical column (Wilson and Cowan, 1972; Eggert and van Hemmen, 2000). The state of each cell *x* is uniquely defined by its membrane potential *V(x,t)*, specified by the following equation:

(1.1) $$\tau \;\cdot \;\frac{dV(x,t)}{dt}=-V(x,t)+k_{1}(V_{In}(x,t)\;+\;k_{2}\eta (x,t))$$

where V_*In*_(*x,t*) (defined by Equation 1.2 below) represents the net input to cell *x* at time *t* (sum of all inhibitory and excitatory postsynaptic potentials—I/EPSPs), τ is the membrane's time constant, *k*_1_, *k*_2_ are scaling values (see Table 1 for the specific parameter values used in the simulations) and η(·,*t*) is a white noise process with uniform distribution over [−0.5,0.5].

(1.2) $$V_{In}(x,t)=-k_{G}\omega_{G}(A_{x},t)+\sum_{\forall y}w_{x,y}\;\cdot \;\phi (y,t)$$

**Table 1:** **Typical parameter values used during the simulations**.

Equation (1.1)	Time constant (excitatory cells)	τ = 2.5 (simulation time-steps)
	Time constant (inhibitory cells)	τ = 5 (simulation time-steps)
	Total input rescaling factor	*k_1_* = 0.01
	Noise amplitude	
	during learning:	*k_2_* = 5 ·$\sqrt{\left(24/\Delta \text{t}\right)}$
	during testing:	*k_2_* = 50 ·$\sqrt{\left(24/\Delta \text{t}\right)}$
Equation (1.2)	Global inhibition strength	
	during learning:	*k_*G*_* = 0.75
	during testing:	*k_*G*_* = 0.60
Equation (2)	Spiking threshold	*thresh* = 0.18
	Adaptation strength	α = 7.0
Equation (3.1)	Adaptation time constant	τ_*ADAPT*_ = 10 (time steps)
Equation (3.2)	Rate-estimate time constant	τ_*Favg*_ = 30 (time steps)
Equation (3.3)	Global inhibition time constant	τ_*GLOB*_ = 12 (time steps)
Equation (4)	Postsynaptic membrane potential thresholds:	
		θ+ = 0.15
		θ− = 0.14
	Presynaptic output activity required for LTP:	
		θ_*pre*_ = 0.05
	Learning rate	Δ = 0.0008

In Equation (1.2) above *y* varies over all cells in the network, *w*_*x, y*_ is the weight of the link from *y* to *x*, and ϕ(*y*,*t*) is *y*'s current output (1 or 0), as defined below (Equation 2); ω_*G*_(*A*_*x*_,*t*) is the area-specific (or “global”) inhibition for area *A* where cell *x* is located (see explanation below and Equation 3.3): this term is identical for all excitatory cells *x* in *A* and absent for inhibitory cells (*k*_*G*_ is a scaling constant). The weights of inhibitory synapses are assigned a negative sign. Note that noise is an inherent property of each model cell, intended to mimic the spontaneous activity (baseline firing) of real neurons. Therefore, noise was constantly present in all areas, in equal amounts (inhibitory cells have *k*_2_ = 0, i.e., the noise is generated by the excitatory cells).

The output (or transformation function) ϕ of an excitatory cell e is defined as follows:

(2) $$\phi (e,t)=\{\begin{array}{l} 1\;if\;V(e,t)-\alpha \omega (e,t)>thresh\\ 0\;otherwise \end{array}$$

Thus, an excitatory cell *e* spikes (= 1) whenever its membrane potential *V*(*e,t*) overcomes a fixed threshold *thresh* by the quantity αω(*e,t*) (where α is a constant and ω, the cell-specific adaptation, is defined below). Inhibitory cells are graded response; the output ϕ(*i,t*) of an inhibitory neuron *i* is 0 if *V*(*i,t*) < 0 and *V*(*i,t*) otherwise.

To simulate spike-rate adaptation (Kandel et al., 2000), function ω(·,*t*) is defined so as to track the cell's most recent firing activity. More precisely, the amount of adaptation ω(*e,t*) of cell *e* at time *t* is defined by:

(3.1) $$\tau_{ADAPT}\;\cdot \;\frac{d\omega (e,t)}{dt}=-\omega (e,t)+\phi (e,t)$$

where τ_*ADAPT*_ is the “adaptation” time constant. The solution ω(*e,t*) of Equation (3.1) is the low-pass-filtered output ϕ of cell *e*, which provides an estimate of the cell's most recent firing-rate history. A cell's average firing activity is also used to specify the network's Hebbian plasticity rule (see Equation 4 below); in this context, the (estimated) instantaneous mean firing rate ω_*E*_(*e*,*t*) of an excitatory neuron *e* is defined as:

(3.2) $$\tau_{Favg}\;\cdot \;\frac{d\omega_{E}(e,t)}{dt}=-\omega_{E}(e,t)+\phi (e,t)$$

In addition to the local excitatory-inhibitory circuits explained in the previous paragraphs (see Figure 1D), mediating local competition mechanisms (Duncan, 1996, 2006), the network also implements an area-specific inhibitory mechanism, which serves the main purpose of keeping the total (“global”) firing activity of excitatory cells in an area within physiological levels (Braitenberg and Schüz, 1998). This mechanism is assumed to be slower than the excitatory-inhibitory dynamics (which typically leads to oscillations in roughly the gamma range), and is realized by a single graded-response unit that estimates the total firing activity within a model area and then, in turn, inhibits all excitatory neurons proportionally (and by the same amount). The area-specific amount of global inhibition ω_*G*_(*A*,*t*) for area *A* at time *t* is defined by Equation (3.3) below:

(3.3) $$\tau_{GLOB}\;\cdot \;\frac{d\omega_{G}(A,t)}{dt}=-\omega_{G}(A,t)+\sum_{e\in A}\phi (e,t)$$

Excitatory links within and between (possibly non-adjacent) model areas are established at random and limited to a local (topographic) neighborhood; weights are initialized independently and at random, uniformly distributed in the interval [0, 0.1]. The probability of a synapse to be created between any two cells falls off with their distance (Braitenberg and Schüz, 1998) according to a Gaussian function clipped to 0 outside the chosen neighborhood (a square of size *n* = 19 for excitatory and *n* = 5 for inhibitory cell projections). This produces a sparse, patchy and topographic connectivity, as typically found in the mammalian cortex (Amir et al., 1993; Kaas, 1997; Braitenberg and Schüz, 1998; Douglas and Martin, 2004).

The Hebbian learning mechanism implemented simulates well-documented synaptic plasticity phenomena of long-term potentiation (LTP) and depression (LTD), as formalized by Artola, Bröcher and Singer (Artola et al., 1990; Artola and Singer, 1993). This rule provides a realistic approximation of known experience-dependent neuronal plasticity and learning (Rioult-Pedotti et al., 2000; Malenka and Bear, 2004; Finnie and Nader, 2012), and includes both (homo- and hetero-synaptic, or associative) LTP, as well as homo- and hetero-synaptic LTD. In the model, we discretized the continuous range of possible synaptic efficacy changes into two possible levels, +Δ and −Δ (with Δ < <1 and fixed). Following Artola et al. we defined as “active” any (axonal) projection of excitatory cell *e* such that the estimated firing rate ω_*E*_(*e*,*t*) of cell *e* at time *t* (see Equation 3.2) is above θ_*pre*_, where θ_*pre*_∈[0,1] is an arbitrary threshold representing the minimum level of presynaptic activity required for LTP to occur. Thus, given a pre-synaptic cell *i* making contact onto a post-synaptic cell *j*, the change Δ*w*(*j,i*) in efficacy of the (excitatory-to-excitatory) link from *i* to *j* is defined as follows:

(4) $$\Delta w(j,i)=\{\begin{array}{l} +\Delta \;if\;\omega_{E}(i,t)\geq \theta_{pre}\;and\;V(j,t)\geq \theta_{+}\;(LTP)\\ -\Delta \;if\;\omega_{E}(i,t)\geq \theta_{pre}\;and\;\theta -\leq V(j,t)<\theta_{+}\\ \;(homosynaptic\;LTD)\\ -\Delta \;if\;\omega_{E}(i,t)<\theta_{pre}\;and\;V(j,t)\geq \theta_{+}\\ \;(heterosynaptic\;LTD)\\ 0\;otherwise \end{array}$$

Furthermore, the implementation of the above rule is subject to the presence, at time-step *t*, of a pre- or postsynaptic spike. In other words, Equation (4) is applied only when the following (inclusive OR) condition holds true:

ϕ(i,t)=1∨ϕ(j,t)=1

where ϕ(·,*t*) is defined by Equation (2). The low-pass dynamics of the cells (Equations 1.1–2, 3.1–3) are all integrated using the Euler scheme with step size Δ*t* = 0.5 ms.

### Simulating learning of meaningful words

We implemented 12 different instances of randomly initialized networks having the structure described above. Initially, each network was in a “naïve” state, in which all synaptic links (both within and between areas) connecting pairs of excitatory cells were established at random, as were their synaptic weights. Word learning and semantic grounding were then simulated by means of repeated learning trials, involving concomitant stimulation of the primary areas of the network.

More precisely, we simulated the learning of six object- and six action-related words. To teach the model an object-related “word,” we repeatedly confronted its primary areas A1, M1i, and V1 with a triplet of pre-defined activation patterns. An activation pattern was simply a set of 19 randomly chosen cells (~3% of the total 25-by-25 cells in one area). This was intended to reproduce a grounded learning situation in which words that are used to speak about visually perceivable objects are acquired via active usage (concomitant activity in A1 and M1i) in presence of the referent object (pattern in V1) (Harnad, 1990; Vouloumanos and Werker, 2009). Similarly, acquisition of an action-related word was simulated by repeated stimulation of areas A1, M1i, and M1_L_, mimicking a situation in which the learning child uses the novel lexical item while executing the corresponding action (Tomasello and Kruger, 1992). Each of the 12 “sensorimotor” patterns was presented repeatedly in 3000 learning trials, resulting in a total of 36,000 (randomly ordered) trials. Each of the 12 network instances was subjected to the same training procedure, using 12 different sets of (six object- and six action-related) sensorimotor “word” patterns. The training procedure is identical to that described in (Garagnani and Pulvermüller, 2016)—the reader is referred to Section “Simulating semantic symbol grounding” in that publication for more details.

### Data collection and analysis

After training, we recorded the network dynamics (responses to “word” and “pseudoword” patterns—see Section Simulated responses to words and pseudowords below—as a function of time). Responses were collected separately for the 12 network instances, for each of the 12 areas, and in case of word stimuli, for each semantic category (action- and object-related words).

#### Simulated responses to words and pseudowords

Each trained network was confronted with an “auditory” activation pattern to area A1 for 500 ms (= 1000 simulation time-steps), simulating perception of a speech sound. The stimulus was either one of the “familiar,” learned word patterns, or an “unfamiliar,” untrained pseudoword pattern. Pseudoword patterns were built by randomly recombining sub-parts of the word patterns used for the training. More precisely, for each network, the “auditory” component of each word pattern (presented to area A1 during training) was divided into 25 parts, consisting of 5-by-5 squares of 25 cells each; the “sub-squares” from the 12 word stimuli were then randomly recombined—preserving their spatial position—to form 12 novel pseudoword stimuli (each containing 2 sub-squares from each of the original word patterns)^1^.

Each testing trial started with a global network reset, upon which the membrane potential of all excitatory and inhibitory cells was set to 0. An interval of 1.5 s (equivalent to 3000 simulation-time steps) followed, during which no input was provided and the network's activity was driven by noise (simulating spontaneous baseline firing). The stimulus was then presented to area A1 for 500 ms, followed by noise again during an inter-stimulus interval of 1 s (total trial length was 3 s). Each stimulus was presented for 10 repeated trials, leading to a total of 240 testing trials (corresponding to 12 min “real time,” or 1,440,000 simulation time-steps) per network. During each testing trial we recorded network activity (total number of spikes and sum of all excitatory cells' membrane potentials in each area at each simulation time-step). In the remainder of the article, we refer to the network's responses in each testing trial (sum of all excitatory cell's membrane potentials in each area) as to the “simulated event-related potential” (S-ERP) responses.

### Data processing for time-frequency and synchronization analysis

To investigate presence, power, and synchrony of oscillatory activity in the network we analyzed the dynamic responses using Morlet wavelet analysis (Tallon-Baudry et al., 1997; Herrmann et al., 2005; Roach and Mathalon, 2008). More precisely, single-trial S-ERPs from each network area were convolved with a six-cycle Morlet wavelet (number of cycles *c* = 6; wavelet length *m* = 3; normalization factor $A={\sigma_{t}}^{{-}^{1}{/}_{2}}\;\pi^{{-}^{1}{/}_{4}}$) in 1 Hz and 10 ms bins from 4 to 100 Hz on the whole trial length (3 s). The resulting single-trial total spectral power was then averaged across trials and networks, separately for pseudoword and word items, and (when appropriate) for semantic category (object- and action-related word). The same Morlet wavelet time-frequency decomposition was applied also to each network's averaged S-ERPs (obtained as described in the previous Section), thus resulting in an estimate of the evoked spectral power (time- and phase-locked). An estimate of the induced spectral power (time-locked but not phase-locked) was then obtained by subtracting the evoked power from the averaged total power (Tallon-Baudry and Bertrand, 1999; David et al., 2006; Roach and Mathalon, 2008) in each condition. Baseline correction was performed by subtracting average activity between −500 and −100 ms (Roach and Mathalon, 2008).

To quantify the degree of between-area synchrony in the different conditions we analyzed the coherence of the single trials' complex wavelet coefficients; this measure is commonly taken as an index of the synchronous activity between different recording sites (Herrmann et al., 2005; Roach and Mathalon, 2008; Sankari et al., 2012; Bastos and Schoffelen, 2015). More precisely, the coherence of the oscillatory activity between the articulatory motor area (M1i), at one “end” of the network, and the primary visual (V1) and dorsal motor (M1_L_) areas, at the other “ends,” was calculated separately for each word category and network instance, and averaged across the 12 networks. We expected coherence between M1i (used as seed channel) and primary areas V1, M1_L_ to differ depending on the semantic category (action- vs. object-related items) of the word stimulus. For the above processing steps we used the Fieldtrip toolbox (Oostenveld et al., 2011) for Matlab (The Mathworks, Natick, MA, USA).

### Statistical analysis

In order to compare spectral power induced in the network by word and pseudoword stimuli, a two-tailed cluster-based permutation statistics with 1000 permutations and a *t*-test for dependent samples as thresholding statistics was carried out across all 12 areas and all 10-ms time bins of the epoch (from −1.5 to 1.5 s) on the average spectral power in the *a priori* selected frequency range between 20 and 40 Hz. The cluster-based permutation procedure is a non-parametric statistical test that controls the false alarm rate due to multiple comparisons of multidimensional data and is widely used for analyzing time-frequency data (Maris and Oostenveld, 2007). The analysis was performed using the Fieldtrip toolbox (Oostenveld et al., 2011) for Matlab (The Mathworks, Natick, MA, USA).

## Results

**Figure 2:**
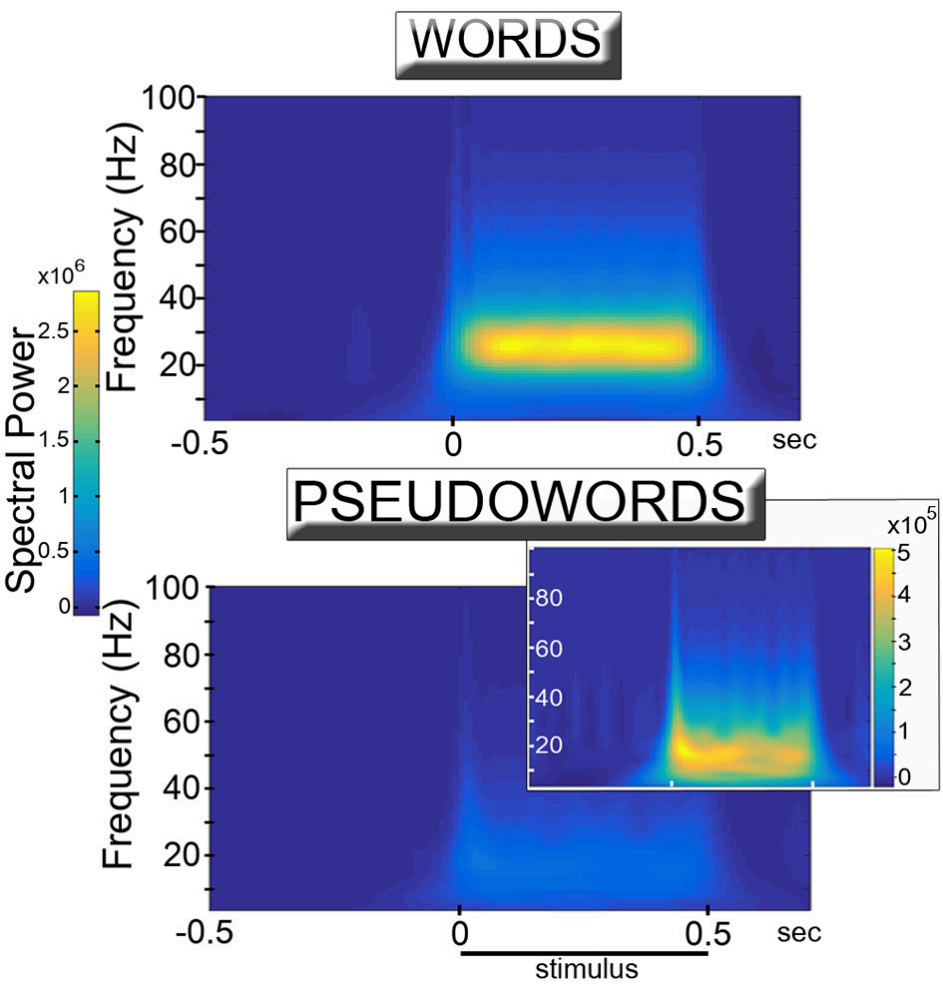
**Simulated high-frequency responses to familiar, meaningful word (Top)** and unknown, meaningless pseudoword (**Bottom** & Inset) items. The diagrams plot induced power (averaged across 12 network areas and 12 network instances) of the network's event-related responses (see Figure 3) in the different frequency bands as a function of time. Learned, familiar word patterns induce strong oscillatory responses in the lower gamma band (25–30 Hz) during stimulus presentation. Unknown pseudoword stimuli appear to induce significantly weaker high frequency spectral responses. Adequate rescaling of the diagram plotting pseudoword responses (Inset) uncovers the presence of (smaller amplitude) oscillatory phenomena which, however, appear to peak at somewhat lower (~20 Hz) frequencies.

The training of the network led to the spontaneous formation of cell assembly circuits analogous to those obtained in previous (non-spiking) versions of the architecture (Garagnani and Pulvermüller, 2016; Tomasello et al., 2016), that is, sets of strongly and reciprocally connected cells linking together correlated patterns of “sensorimotor” activity. Visual observation of the network responses during presentation of learned “word” and novel “pseudoword” items to the model correlate of primary auditory cortex indicated that both types of stimuli induced oscillatory phenomena, manifest in the form of “pulses” or waves of activity propagating across the network. Quantitative analysis of the recorded simulated responses confirmed this observation, but also revealed strong differences between the responses in the two conditions. Figure 2 reports the induced power in response to word and pseudoword presentation. The plots show a clear difference between the two conditions, particularly evident in the lower gamma band (25–30 Hz). Results of the statistical analysis fully confirmed this: the cluster-based permutation test comparing word vs. pseudoword responses in the 20–40 Hz frequency range revealed a significant difference between the two conditions (*p* = 0.0001). The positive cluster indicating higher spectral power for words than pseudowords extended over all areas and over the interval from −50 to 550 ms, corresponding to stimulus duration (considering the minimal time uncertainty intrinsic to time-frequency decomposition).

As the observed changes in (average) spectral power could be explained by changes in either the degree of synchronization of the signals across different trials or in the magnitude of the oscillations (or both) (Roach and Mathalon, 2008), in order to estimate whether word and pseudoword stimuli induced different magnitude oscillations we ran an additional analysis using the peak membrane potential value reached (within an area) during the 50-to-500 ms interval of each trial, averaged across trials and network areas separately for the two conditions. A paired-sample *t*-test on these data confirmed that words exhibited larger peak amplitude responses than pseudowords [*t*_(11)_ = 4.3, *p* = 0.001].

**Figure 3:**
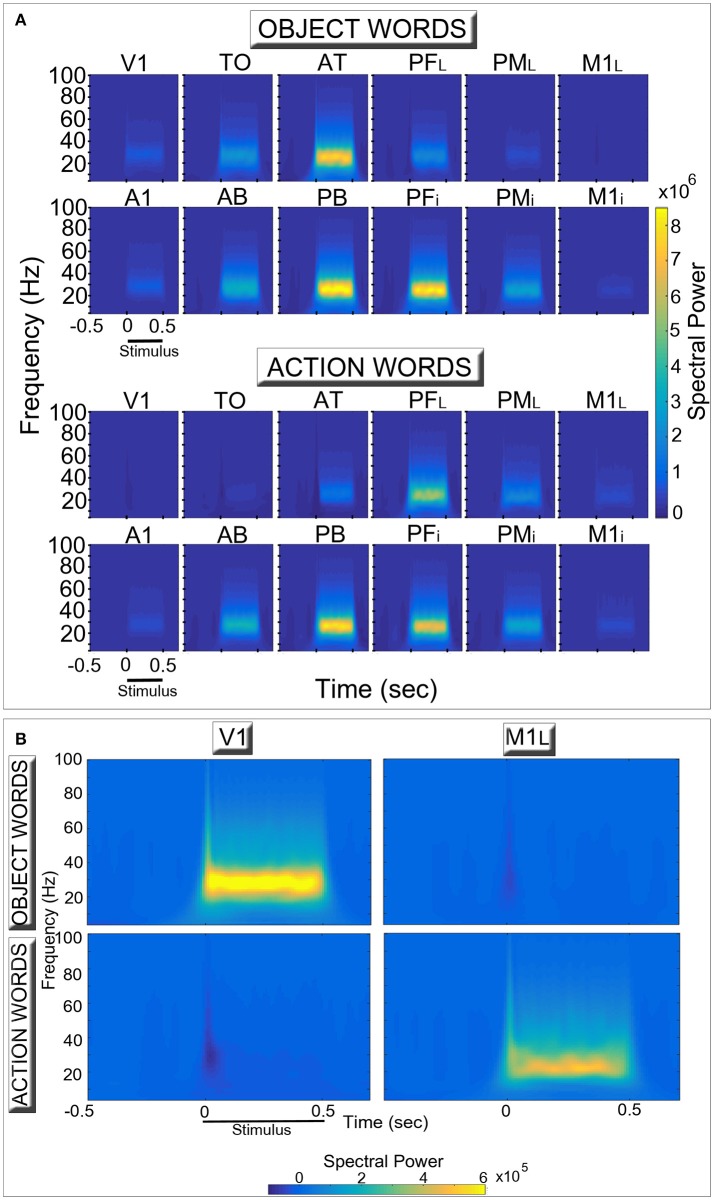
**Network oscillatory responses during presentation of familiar object- (Top row in each diagram) and action-related (Bottom row) words. (A)** for each area, induced spectral power of the simulated event-related responses is plotted for the different frequency bands and two conditions as a function of time. **(B)** a rescaled version of **(A)**, plotting only data from the two primary extrasylvian areas (V1, M1_L_). During presentation of a stimulus to area A1, both word categories induced high-frequency oscillatory activity peaking between 25 and 30 Hz (in line with the across-area averages shown in Figure 2, top plot) which appear stronger in the central areas (AT, PF_L_, PB, PF_i_) **(A)**. Note the double dissociated responses exhibited by the extrasylvian areas (V1, TO, AT, PF_L_, PM_L_, M1_L_). In particular, category-specific oscillations emerge in primary visual and motor areas **(B)**, with the former (V1) selectively responding to object-related words and the latter (M1_L_) to action-related ones. Also note the presence of oscillatory responses at frequencies higher than 30 Hz.

Figure 3 shows induced spectral power for the two semantic categories (action- and object-related words) in the different network areas. The time-frequency decomposition reveals topographically distinct spectral responses in the six extrasylvian areas (top lines of each diagram in Panel A) for the two word categories, particularly evident in the two “hub” areas (AT, PF_L_). By contrast, the patterns in the six perisylvian areas (bottom lines) do not appear to exhibit between-category differences.

**Figure 4:**
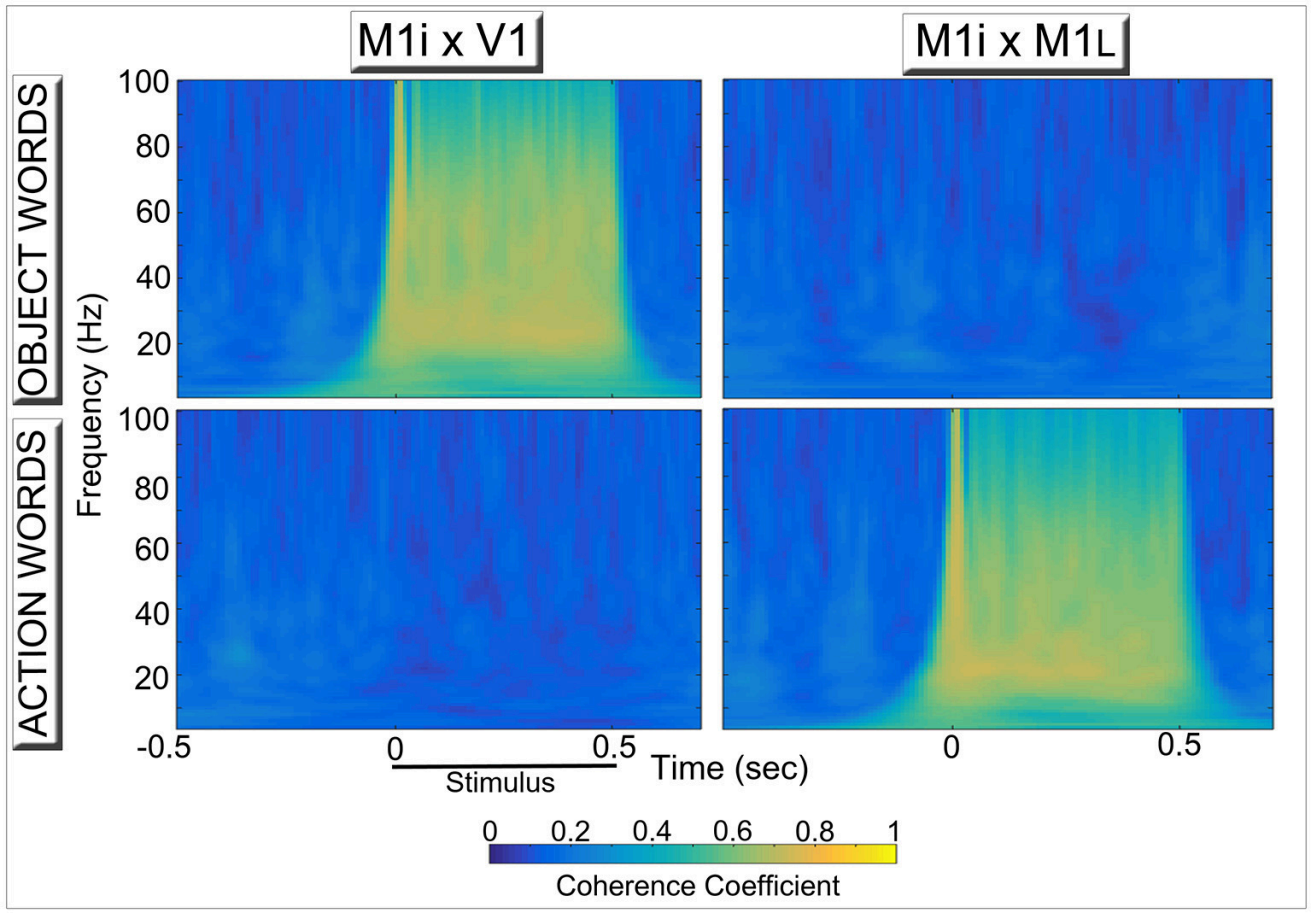
**Synchronous activity in primary visual (Left)** and primary motor **(Right)** areas induced by simulated recognition of spoken words grounded in the context of visual perception **(Top)** and action execution **(Bottom)**. Coherence coefficients between oscillatory responses in area M1i (where CA-circuit parts conveying model correlates of “articulatory” information are reactivated) and primary visual (V1, Left) and motor (M1_L_, Right) areas (where simulated “perception” and “action” patterns of activation, respectively, are stored) during presentation of object- and action-related words to area A1 are plotted for the different frequency bands as a function of time. The synchronous activity reflects the periodic spreading of activity waves within stimulus-specific CA circuits (see Figure 2, top plot), which link up phonological patterns in “auditory-articulatory” areas (A1, M1i) with “semantic” information coming from the model's sensory (V1) or motor (M1_L_) systems. Note the clear double dissociation, whereby “articulatory” areas show a high degree of synchronization with “visual”—but not with “motor”—areas during presentation of words with object-related meaning (Top diagrams) and action-related words exhibit the opposite pattern (Bottom diagrams), mirroring the spectral data shown in Figure 3B.

Figure 4 reports results of the coherence analysis performed on the oscillatory responses from three of the 12 network areas during simulated word recognition processes (data plotted in Figure 3B). More precisely, the degree of synchrony between oscillations in area M1i and either primary visual (V1, left) or primary motor (M1_L_, Right) areas induced by presentation of learned, meaningful object- and action-related words to area A1 is plotted as a function of time. Note the category specific double dissociation of synchronous oscillations exhibited by even “distant” (i.e., more than 1 synaptic step away) network areas.

## Discussion

**Figure 5:**
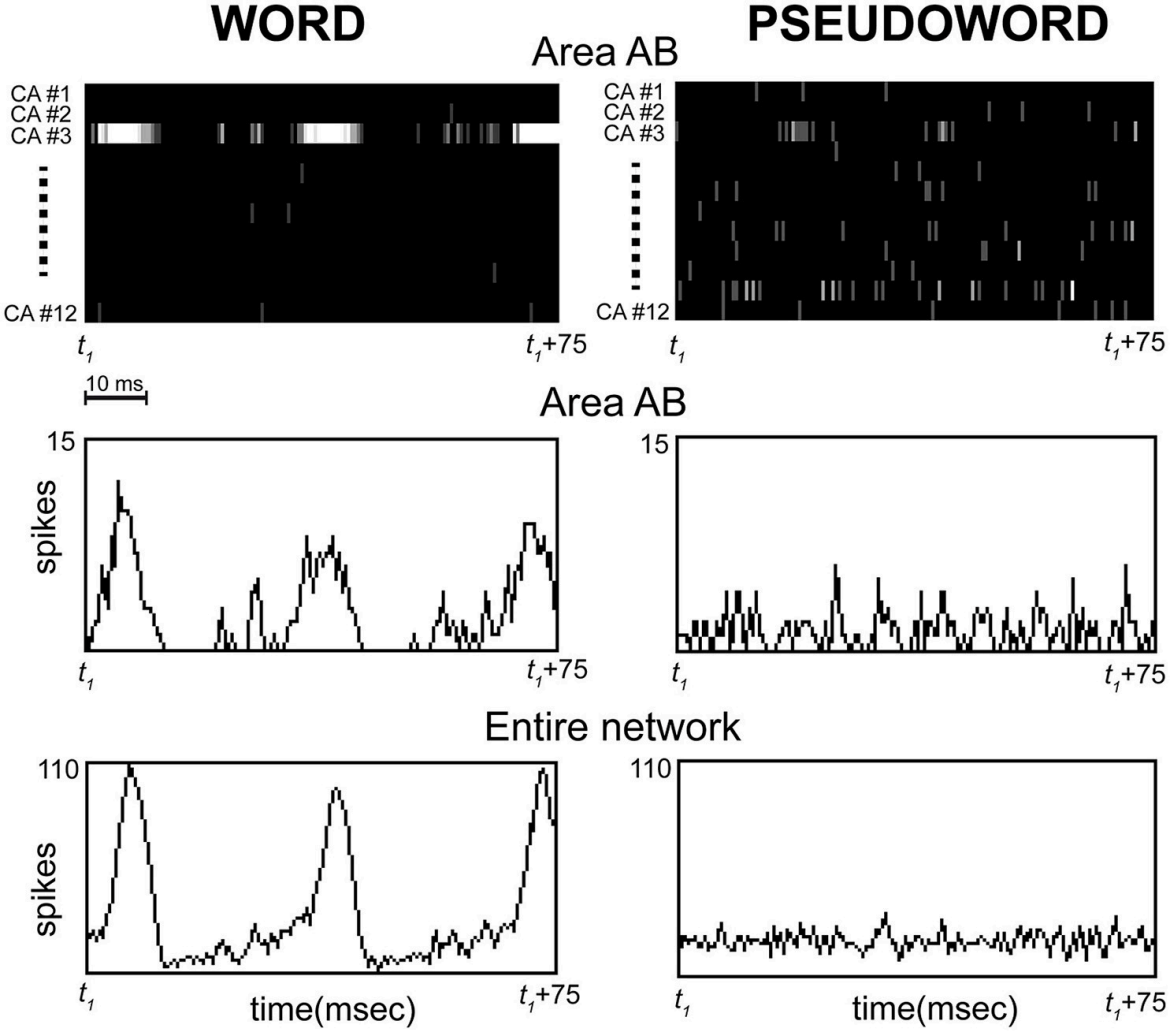
**Representative example of simulated spiking responses over a 75 ms interval sampled during continuous stimulation of area A1 with one of the learned word (Left) or “unknown” pseudoword (Right) patterns**. The initial time point of the interval (*t1*) does not represent stimulus onset, but an arbitrary time point chosen after the network had reached the “steady state.” Top: CA-specific raster plots showing spiking activity within each of the 12 cell assemblies (one for each line, labeled CA#1,…CA#12) in a representative network area (AB) are reported for the two conditions as a function of time; spikes are depicted as vertical lines on the black background (brightness indicates number of spikes per time bin). Middle: histograms plotting the total number of spikes per time bin in area AB for the two conditions as a function of time. Bottom: as Middle, but the histograms plot the total number of spikes within the entire network. Note (Left) the strong oscillatory activity (spike waves of ~30 ms period) emerging selectively within CA-circuit #3 during stimulation with the corresponding word pattern, and (Right) the absence of such strong responses during pseudoword presentation, which is characterized instead by similar firing rates across all CA circuits and irregular, “out-of-synch” activity peaks (e.g., see CA#3 and CA#11). Also note the synchrony between the oscillations occurring in all network areas during word stimulation, suggested by the alignment between the peaks of the spike waves in the histograms for area AB (Middle-left) and entire network (Bottom-left). Time bins were 0.5 ms.

We implemented a biologically realistic, spiking neural-network architecture closely replicating anatomical connectivity and cortical features of primary, secondary, and higher-association areas in the frontal, temporal, and occipital lobes of the human brain, and applied it to investigate the neural mechanisms underlying differential oscillatory responses to meaningful action- and object-related words and novel, senseless pseudoword stimuli. As a result of the simulated process of word learning, we observed the emergence of distributed, stimulus-specific cell-assembly circuits, binding phonological (acoustic-articulatory) patterns in perisylvian areas with co-occurring semantic information coming from the sensory and motor (extrasylvian) systems. Crucially, after cell-assembly circuit emergence, the presentation of a learned “word” stimulus to the model correlate of primary auditory cortex (area A1) induced coherent oscillatory activity in the network within the lower gamma band (25–30 Hz), manifest as periodic “pulses” (spike bursts) of activity occurring within the cell-assembly circuit specific to that stimulus (see Figures 2, 3, 5). By contrast, presentation of a novel, unfamiliar “pseudoword” pattern led to significantly smaller-amplitude oscillatory responses. These findings are consistent with experimental results reporting larger gamma band responses to words than pseudowords (Lutzenberger et al., 1994a,b; Pulvermüller et al., 1994, 1996a; Krause et al., 1998; Mainy et al., 2008). Furthermore, the cortical topography of stimulus-induced oscillatory patterns exhibited clear dissociations between semantic word categories in terms of both local spectral power (Figure 3) and inter-area coherence (Figure 4), again in agreement with some pre-existing experimental reports (Pulvermüller et al., 1996b, 1999; Weiss and Müller, 2013). These results, documenting category-specific spreading of activity within the stimulated CA circuits, provide a neuromechanistic account of action- and object-related word learning and recognition in the brain, as discussed below in light of neurophysiological evidence.

### Mechanisms underlying the enhancement of the simulated high-frequency responses to words vs. pseudowords

In order to understand the model mechanisms that led to the observed result, we inspected the network's dynamic behavior directly during stimulation. This revealed that, unlike words, pseudoword stimuli do not induce activation specifically within a single CA circuit, but, instead, partial co-activation of many cell-assembly circuits, within which smaller-amplitude^2^, sub-threshold, oscillatory activity occurs (see Figure 5). To understand why this is so, note that each pseudoword pattern was built by randomly combining smaller sub-parts of the “learned” word patterns; therefore, presentation of a pseudoword conveys an equal amount of activity (on average) in all word circuits at once. This activity, however, is significantly lower (~1/12) than the amount conveyed into a single CA circuit by a word pattern. In addition, the presence of regulatory mechanisms in the network (i.e., area-specific inhibition) leads the simultaneously stimulated circuits to inhibit each other; this reciprocal suppression (or “competition”) causes anti-phasic activity waves within them, i.e., out-of-synch spike bursts. As a result, the oscillations within different circuits tend to “balance” each other out, leading to smaller-amplitude network responses (note the flat profile of the histograms on the right-hand side of Figure 5).

By contrast, presentation of a learned word pattern conveys the full amount of sensory input into neurons that belong to a single—and hence, “non-competing”—CA circuit; this induces above-threshold activity and thus periodic circuit ignitions, manifest as synchronous bursts (or “waves”) of spikes spreading within the entire circuit and network (Figure 5, Left). To sum up: a word stimulus conveys above-threshold activity within a single cell-assembly, inducing periodic, large-scale and synchronous bursts of activity within it at its spontaneous (“resonance”) frequency; by contrast, pseudoword stimulation induces sub-threshold and “out-of-phase” activity within competing CA circuits, resulting in significantly weaker oscillatory responses.

This result is consistent with our previous simulations, in which we replicated and explained differences in neurophysiological responses to word and pseudoword items (Garagnani et al., 2008). Such simulations showed that, in presence of sufficiently high levels of area-specific (global) inhibition (the model correlate of “low attention”), network responses to familiar, learned “words” are larger than to novel, unknown “pseudoword” stimuli; this was a consequence of the competitive interactions (mediated by area-specific inhibitory loops) occurring between the different CA circuits concomitantly (but only partially) activated by a pseudoword. In the present simulations, relatively high levels of baseline noise (simulating spontaneous neuronal firing) produce similarly strong amounts of global inhibition.

Closer inspection of the results of the time-frequency analysis of the S-ERP data reveals the presence of another difference, namely, in the spectral profile of the responses: while word presentation elicits consistent, strong oscillations around 25–30 Hz during stimulus presentation, the less regular pseudoword-induced responses exhibit power peaks mostly below 20 Hz (see Figure 2, Inset). We hypothesize that the above-mentioned competitive interactions may also underlie this “shift” toward lower-frequencies: in fact, mutual inhibition between co-activated CA circuits likely induces not only smaller responses but also “delays” in the accumulation and propagation of activity within the CA circuits, leading to longer time intervals between the periodic bursts of activity, and hence, to oscillations having generally longer wavelength. The fact that the power of the induced oscillations should peak at lower frequencies for pseudowords than for words is a novel prediction emerging from the model, which, to the best of our knowledge, no other computational account of language processing has generated; experimental data confirming this prediction would therefore provide strong evidence in support of the present mechanistic model.

### Increased spectral power and long-range synchronization during word recognition

#### Spectral power

During presentation of a word stimulus the network exhibited substantial increase in spectral power peaking at around 25–30 Hz (see Figure 2) which, as revealed by Figure 4, had category-specific topographic profile (as predictable from the double dissociations shown by the data plotted in Figure 3). These results are remarkably in line with some of the existing neurophysiological data. In particular, analogous double dissociations in high-frequency spectral power in occipital (visual) and central (motor) recording sites had been found for (visually presented) nouns and verbs having strong visual and motor semantic associations, respectively (Pulvermüller et al., 1996b, 1999). As nouns and verbs differ not only in action-relatedness but also in lexical category, these results were prone to alternative interpretations, due to this confounding factor; more recent evidence (Moseley and Pulvermüller, 2014), however, has revealed differential brain activation to concrete nouns vs. concrete verbs, but not between abstract ones, corroborating the view that word meaning, rather than lexical category, is driving the observed topographical differences in brain responses (Moseley and Pulvermüller, 2014).

More generally, a large number of studies have documented increases in gamma-band response (GBR) amplitude during processing of meaningful words (compared to baseline) (e.g., Canolty et al., 2006; Edwards et al., 2010; Pei et al., 2011; Wu et al., 2011; Vignali et al., 2016). Most relevant to the present results, higher spectral power during processing of familiar items (words) vs. unfamiliar ones (pseudowords or non-words) has been found in English using MEG (Pulvermüller et al., 1996a) and ECoG (Canolty et al., 2007), in Finnish with EEG (Krause et al., 1998), in German with MEG (Eulitz et al., 1996), and in French, using intracortical recordings (Mainy et al., 2008), with remarkable consistency across languages, sensory modalities, and recording methods.

#### Long-range (“inter-area”) synchronization

The network simulations revealed a high degree of synchronization between model areas that are only indirectly connected (in particular, M1i–V1, top-left of Figure 4, and M1i–M1_L_, bottom-right of Figure 4); crucially, such long-range synchrony depended on the semantic category, and was a by-product of the dynamic activation of circuits that included (or lacked) functional links between articulatory-phonological (M1i) and stimulus-specific semantic information in either primary motor (M1_L_) or visual perceptual (V1) areas.

Experimentally, between- (inter-) -area synchronization of oscillatory activity in non-adjacent cortical areas (here referred to as “long-range” synchronization) has been widely documented in different sensory modalities and during different cognitive tasks using both invasive and non-invasive methods (see Varela et al., 2001; Kaiser and Lutzenberger, 2003; Womelsdorf et al., 2007; Buzsáki and Wang, 2012; Harris and Gordon, 2015 for reviews). In particular, studies in the language domain found changes in long-range cortical synchronization during lexico-semantic and syntactic processing (Weiss and Mueller, 2003; Supp et al., 2004; Weiss et al., 2005; Bastiaansen and Hagoort, 2006; Mellem et al., 2013; Weiss and Müller, 2013). Most relevant here is the recent work by Weiss and Mueller (2003), who analyzed oscillatory neurophysiological responses to concrete and abstract spoken words placed in semantically congruent and incongruent contexts. The authors found that, in incongruent sentences, lower-range (29–34 Hz) gamma band coherence between frontal and posterior recording sites was higher for concrete than for abstract items, interpreting this difference as indexing presence and reactivation of lexical-semantic circuits widely distributed over sensory and motor cortices (Weiss and Müller, 2013). We should note, however, that coherence as measured at scalp level cannot be unequivocally attributed to synchronous oscillatory activity in distinct brain sources, due to the presence of possible volume conduction artifacts (Guevara et al., 2005; Trujillo et al., 2005; Bastos and Schoffelen, 2015). Thus, in order to adequately test the prediction emerging from the present simulation results (in particular, Figure 4)—i.e., that word meaning comprehension processes are grounded in primary areas in a category specific manner—further studies of language-induced synchronous oscillations (either by means of intracranial recordings in patients or in source space) are desirable, potentially adopting paradigms successfully used in the past to reveal brain correlates of category specific semantic activations (Carota et al., 2012; Moseley et al., 2012).

### High-frequency cortical responses and long-range synchronization in non-linguistic domains

As the neuroscientific principles (in particular, Hebbian learning) underlying the emergence of word-related memory circuits in the perisylvian areas are putatively at work in all parts of the cortex, this account predicts - and is consistent with experimental evidence indicating the presence of - similar differences in high-frequency responses to familiar, well-learned vs. unknown, unrecognizable items in other modalities, due to the putative emergence of analogous CA circuits there for the commonly occurring percepts. Indeed, different types of gamma oscillations have been documented not only in the auditory, but also visual, olfactory, and somatosensory modalities, as well as during motor tasks, of both humans and animals (Tallon-Baudry and Bertrand, 1999; Engel and Singer, 2001; Cheyne, 2013). In the visual domain, earlier work on basic stimuli, investigating GBRs to coherently (i.e., parallel) vs. incoherently moving bars (Gray and Singer, 1989; Gray et al., 1989; Engel et al., 1991a,b) in animals was closely followed by cognitive investigations, with real object pictures eliciting greater GBRs than pictures of unrecognizable, fragmented or scrambled objects or faces (Tallon-Baudry et al., 1996; Gruber et al., 2002; Henson et al., 2009; Hassler et al., 2011; Bertrand et al., 2013; Gao et al., 2013; Craddock et al., 2015). Although, Yuval-Greenberg and colleagues (Yuval-Greenberg et al., 2008) showed that induced gamma-band activity (iGBA) in neurophysiological data can be contaminated by artifacts originating from miniature saccades or muscle activity, we note that: (1) several of these results can hardly be attributed to effects of microsaccades, as, for example, these studies controlled for the physical features of the stimuli (Gruber et al., 2002), presented stimuli tachistoscopically so that eye movements were discouraged or excluded muscle artifacts based on EMG recordings (Pulvermüller et al., 1997), or used intracortical recording methods (or magnetoencephalography, MEG) (Bertrand et al., 2013; Gao et al., 2013), which are minimally affected by small eye artifacts; (2) some evidence suggests that microsaccades actually decrease when looking at a coherent stimulus as compared to an incoherent one (Makin et al., 2011); and (3) the use of artifact-removing methods such as independent component analysis and beamforming (Keren et al., 2010; Craddock et al., 2016) enables identifying iGBA activity increases in the signal even after removal of miniature-saccade effects (Hassler et al., 2011, 2013; Craddock et al., 2015).

The results that reduced synchronization in the olfactory system can impair odor discrimination (Stopfer et al., 1997; Martin and Ravel, 2014) and that modulation of both gamma and beta responses are linked with changes induced by olfactory learning (Ravel et al., 2003; Martin et al., 2004) also constitute further pieces of evidence in support of the hypothesis mentioned at the beginning, i.e., that CA circuits for commonly occurring percepts may emerge in the cortex in different modalities and cognitive domains.

The results plotted in Figure 5 (in particular, middle and bottom-left diagrams) suggest that, during word presentation, the oscillations in the different model areas exhibit an almost zero time-lag synchronization. The emergence of quasi-zero phase-lag in the simulations is interesting, but not entirely surprising: previous work using multi-area spiking networks has linked this phenomenon, for example, to local inhibitory interactions (Traub et al., 1996) or global regulatory loops (Vicente et al., 2008), both of which are implemented here. It is known, however, that modeling realistic axonal transmission delays may also prevent zero-lag synchronization, or even induce anti-phase interactions (Knoblauch and Palm, 2002; Knoblauch and Sommer, 2003); as the present model does not implement conduction delays, any strong prediction about the phase lag based on the results presented here should be taken with caution (Viriyopase et al., 2012). On the other hand, experimental evidence for zero time-lag synchronization across distant cortical regions (including interhemispheric areas) and sensory modalities during different tasks has been observed, using invasive recordings in both humans—typically from epileptic patients in surgical settings (e.g., Rodriguez et al., 1999; Lachaux et al., 2005)—and animals, in the beta (Bressler et al., 1993; Roelfsema et al., 1997; Witham et al., 2007) and gamma band (Engel et al., 1991b; Roelfsema et al., 1997; von Stein et al., 2000; Gregoriou et al., 2009). Note that the role of synchrony and neural-population responses in cognition is object of ongoing research (Gilad and Slovin, 2015; Martin and von der Heydt, 2015).

### Summary

We present a spiking, neuroanatomically realistic neural-network model able to simulate and explain larger high-frequency neurophysiological responses to familiar words than novel, unknown pseudoword stimuli on the basis of spontaneous emergence and competitive interactions of cell-assembly circuits for words. The model links the different spectral responses to corresponding differential oscillatory dynamics of underlying large-scale neuronal populations, with periodic “bursts” of spikes occurring within a single, stimulus-specific circuit during presentation of a well-learned, meaningful word, and absence thereof during pseudoword input (characterized, instead, by “out-of-phase” and smaller amplitude responses within multiple competing CA circuits). In addition, the model replicates and extends previous results obtained with a simpler, graded-response version of the architecture, demonstrating spontaneous emergence of stimulus-specific cell-assembly circuits by means of a novel, spike-driven Hebbian plasticity rule at work within a more accurate neuroanatomical structure. Finally, in line with existing experimental results, coherence analysis of the simulated neurophysiological responses reveals the presence of double dissociations in the category specific patterns of synchronous oscillations observed in distant cortical areas. Linking cellular-level mechanisms and neuronal-population behavior with cognitive function, this study contributes to bridging the gap between experimental data and scientific theory by means of a computational architecture based entirely on neurobiologically realistic principles, hence providing further evidence in support of an account of word acquisition and semantic learning grounded in action and perception.

## Author contributions

MG and GL conceived the study, conducted the experiments, analyzed the data, wrote the paper. RT contributed to the experiments. TW and FP supervised the study and contributed to paper writing. The first two authors contributed equally to this work.

### Conflict of interest statement

The authors declare that the research was conducted in the absence of any commercial or financial relationships that could be construed as a potential conflict of interest.

## Footnotes

^1^Pseudoword patterns used only 24 of the 25 possible sub-squares of the 625-cell grid. Also note that pseudowords somehow belonged to both of the two semantic categories, as they were made up of a random mix of sub-squares taken from both “action” and “object” words.

^2^This is confirmed by the analysis of the within-trial S-ERP peaks, see Section Results.

## Appendix A

### Additional evidence in support of the model's connectivity structure

Neuroanatomical evidence shows that adjacent cortical areas tend to be connected with each other through next-neighbor between-area connections (Pandya and Yeterian, 1985; Young et al., 1994, 1995). These exist within each triplet of areas of the four domain-specific “sub-systems” modeled, that is, amongst (I) inferior-frontal areas in the articulatory system PF_i_–PM_i_–M1i, (II) superior-lateral areas in the “auditory” system A1–AB–PB (Pandya, 1995; Kaas and Hackett, 2000; Rauschecker and Tian, 2000), (III) superior-lateral frontal areas in the “hand-arm” motor system PF_L_–PM_L_–M1_L_ (see also Arikuni et al., 1988; Lu et al., 1994; Dum and Strick, 2002, 2005), and (IV) inferior temporo-occipital areas in the “visual” system V1–TO–AT (Distler et al., 1993; Nakamura et al., 1993).

Evidence also indicates the presence of long-distance cortico-cortical links (see thicker purple arrows in Figure 1B) connecting areas distant from each other. Amongst the long-distance cortico-cortical links within fronto-temporo-occipital cortex, we implemented only the well-documented mutual and reciprocal connections between anterior temporal (AT), superior parabelt (PB), and inferior (PF_i_) and superior-lateral (PF_L_) prefrontal areas. The connections between inferior anterior (and middle), superior temporal (AT, PB in Figure 1B) and inferior prefrontal (and premotor) cortices (PF_i_) are realized by the arcuate and uncinate fascicles (Makris et al., 1999; Romanski et al., 1999b; Petrides and Pandya, 2001; Makris and Pandya, 2009; Catani et al., 2005; Parker et al., 2005; Romanski, 2007; Rilling et al., 2008; Makris and Pandya, 2009; Petrides et al., 2012; Rilling, 2014). Dorsolateral prefrontal (and premotor) cortex (PF_L_) is reciprocally linked to anterior and inferior temporal regions (AT) via the uncinate fascicle (Kuypers et al., 1965; Pandya and Barnes, 1987, p.49; Ungerleider et al., 1989; Eacott and Gaffan, 1992; Webster et al., 1994) as well as to the superior temporal cortex (PB) via the extreme capsule (Pandya and Barnes, 1987, p.48; Romanski et al., 1999a,b; Schmahmann et al., 2007).

Lastly, links between inferior and superior prefrontal areas (PF_i_–PF_L_) (Yeterian et al., 2012) and between auditory parabelt and anterior temporal cortex (PB–AT) (Gierhan, 2013) were also implemented, as in a recent (graded-response) version of the architecture (Tomasello et al., 2016).
